# Robust Sex Differences in Jigsaw Puzzle Solving—Are Boys Really Better in Most Visuospatial Tasks?

**DOI:** 10.3389/fnbeh.2017.00194

**Published:** 2017-10-23

**Authors:** Vid Kocijan, Marina Horvat, Gregor Majdic

**Affiliations:** ^1^Faculty of Computer and Information Science, University of Ljubljana, Ljubljana, Slovenia; ^2^Department of Psychology, Faculty of Arts, University of Maribor, Maribor, Slovenia; ^3^Institute for Preclinical Sciences, Veterinary Faculty, University of Ljubljana, Ljubljana, Slovenia; ^4^Institute of Physiology, Medical Faculty, University of Maribor, Maribor, Slovenia

**Keywords:** children, sex difference, visuo-spatial tasks, jigsaw puzzle

## Abstract

Sex differences are consistently reported in different visuospatial tasks with men usually performing better in mental rotation tests while women are better on tests for memory of object locations. In the present study, we investigated sex differences in solving jigsaw puzzles in children. In total 22 boys and 24 girls were tested using custom build tablet application representing a jigsaw puzzle consisting of 25 pieces and featuring three different pictures. Girls outperformed boys in solving jigsaw puzzles regardless of the picture. Girls were faster than boys in solving the puzzle, made less incorrect moves with the pieces of the puzzle, and spent less time moving the pieces around the tablet. It appears that the strategy of solving the jigsaw puzzle was the main factor affecting differences in success, as girls tend to solve the puzzle more systematically while boys performed more trial and error attempts, thus having more incorrect moves with the puzzle pieces. Results of this study suggest a very robust sex difference in solving the jigsaw puzzle with girls outperforming boys by a large margin.

## Introduction

Sex differences in different cognitive domains are often discussed, both scientifically and in lay public. The main debates are concerning the actual existence of such differences in different tasks, and, whether these differences are biologically predetermined by sex differences in the brain, or are arising due to differences in upbringing between girls and boys (nature vs. nurture debate). One of the main criticisms of sex differences in behavior is often small difference between sexes (Miller and Halpern, 2014). Indeed, even in most broadly studied tasks like mental rotation task, the differences in success are usually not more than 10% between men and women. For example, a very large study including over 90,000 women and over 110,000 men found consistent sex differences in mental rotation ability and line angle judgment, although the absolute difference was rather small (Lippa et al., 2010) and often, individual variation within one sex is almost as large as the difference between sexes (Miller and Halpern, 2014).

Sex differences in visuospatial abilities are in general acknowledged, with men performing better on mental rotation tests and tests of spatial perception and orientation (Voyer et al., 1995; Halpern et al., 2007; Lippa et al., 2010), while women outperform men in test of memory for object locations (Ecuyer-Dab and Robert, 2004). The origin of these sex differences is not fully understood and different studies suggest different causes. Evolutionary theorists propose that selection pressure on early human populations led to sex differences in spatial abilities, according to their roles in the society (Jones et al., 2003). Some studies suggest that sex difference in visuospatial abilities could be caused by exposure to sex hormones (Halpern et al., 2007; Hines, 2011), while other studies suggest that all these differences arise from differences in social environments and gender socialization (Wood and Eagly, 2002). There is no final answer in regard to the origin of sex differences in these behaviors, partially due to the fact that it is impossible to test these theories in healthy humans, while animal models are unquestionably insufficient and cannot provide full answers in regard to cognitive abilities. Nevertheless, numerous studies with human subjects, exposed to inappropriate levels of sex steroid hormones (Hines, 2011; Miller and Halpern, 2014), and studies in non-human primates (Wallen and Hassett, 2009), showing some similarities in certain sexually differentiated behaviors, suggest an underlying biological cause for these sex differences.

Generally it is considered that men are better in visuospatial tasks, although these conclusions are mostly based on mental rotation tasks and spatial orientation (Lippa et al., 2010; Miller and Halpern, 2014). Interestingly, there are numerous references indicating that adult men perform better than women in jigsaw puzzle solving (Caplan and Caplan, 1997; Helgeson, 2016), but there is no scientific evidence for these statements.

Therefore, in the present study, we tested the hypothesis that there is a sex difference in jigsaw puzzle solving task with males outperforming females.

## Materials and Methods

**Figure 1:**
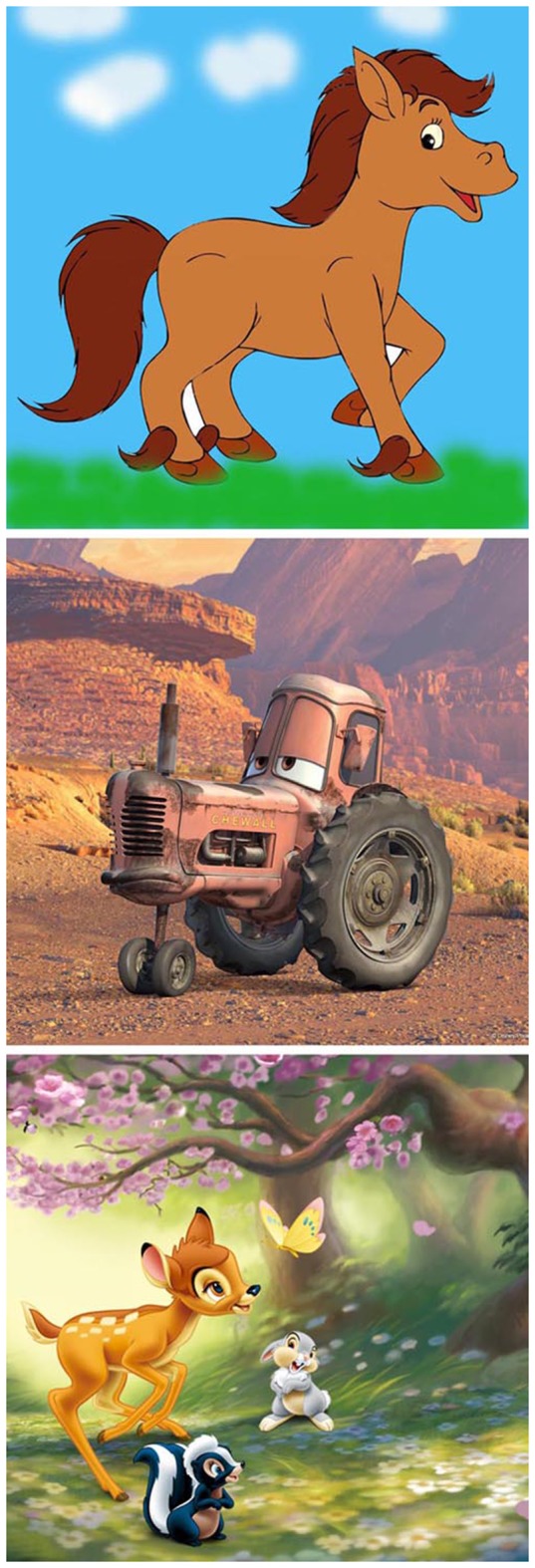
Themes of the three puzzles.

Jigsaw application for Android operating system was prepared especially for this study by VK. The puzzle was square consisting of 25 interlocking pieces (5 × 5 pieces). The application had three different pictures, horse, tractor and Bambi cartoon character (Figure 1). Pictures were chosen as being more sex neutral (horse), more attractive and interesting to boys (tractor) or more interesting and attractive to girls (Bambi). Each participant solved each of the three puzzles in the same sequence (Horse, Tractor, Bambi).

The study was approved by the institutional ethical committee (Committee for Ethics in research, Faculty of Arts, University of Maribor) and all parents of participating children signed an informed consent and agreed that their children could participate in the study. Participation in the study was completely voluntary.

Study was performed in one primary school with children from similar social and economical background. Children participating in the study were all 2nd grade students, born between December 2008 and December 2009. Study was performed in November 2016, so children age was between full 7 years and full 8 years (84–96 months). In total, 24 girls (age 103.45 months ± 0.86, mean ± SEM) and 22 boys (age 103.27 months ± 0.97) participated in the study. None of the participants was diagnosed with any learning deficits or psychiatric disorders, and all were average students for their grade.

None of the kids had seen the puzzle before and had no specific training before the start of the study. During the study, each child was given the tablet with a brief explanation how to solve the puzzle, and was then left alone without any disturbances until successful completion of jigsaw puzzle. Experiment was carried out individually with every child in a separate quiet room in the school.

All data were anonymized with regard to the name of the child when entered into the application, while the application recorded month and year of birth. Application also recorded all movements of the jigsaw pieces. Specifically, parameters recorded were:

1. Total time needed for the completion of puzzle (**Total time**).
2. Total number of wrong moves—i. e., a move when the student took a piece and tried to put it in the wrong place, and released it as it didn’t fit (**Wrong moves**).
3. Total time student hold the piece and moved it around the tablet, trying to find its correct position (**Moving time**).
4. Average time spent moving the pieces around (average time for each piece student was actively holding or moving it around the tablet; **average moving time**).
5. Total time spent just watching the puzzle, without moving or touching pieces (**watching**).

### Statistical Analyses

All results were analyzed using NCSS software package (NCSS, Kaysville, UT, USA). Differences between different themes of the puzzles were analyzed by two-way ANOVA with theme of the puzzle (tractor, horse, Bambi) and sex as independent variables. Preliminary analysis showed no interaction between sexes and the theme of the picture, therefore, the results for each of three different puzzles were averaged and used for further group comparisons. Differences in different parameters between girls and boys were analyzed using ANOVA followed by Bonferroni multiple comparison test. Size effect (eta squared, *η*^2^) was calculated by following formula: *η*^2^ = SS_effect_/SS_total_. Correlation between age and performance on the tasks was evaluated using a linear regression analysis. All differences were considered statistically significant with *p* < 0.05.

## Results

### Correlation between Age and Performance

As children tested were aged between 84 months and 96 months, and there was no statistical significance in the mean age of children, we first checked whether there was any correlation between age and performance on the task. Results revealed no such correlation (*r*^2^ = 0.0236 for time solving and 0.0149 for wrong moves). Therefore, in subsequent analyses, the age was not used as a covariate.

### Difference in Performance and Picture of the Jigsaw Puzzle

To make sure the picture of the puzzle to be solved did not influence the performance on the tasks differently between sexes, ANOVA with sex and theme of the picture as independent variables were used. There was a significant difference in total time needed to solve the puzzle (*p* = 0.027) and number of wrong moves (*p* = 0.008) between themes of the puzzle. In both sexes, the best performance was observed with Bambi picture, which was always the last puzzle children were solving. There was no interaction with sex and it was therefore concluded that this difference occurred as a result of experience training (as Bambi theme was always the last) and not due to the sex difference.

### Sex Difference in Solving Tasks

**Figure 2:**
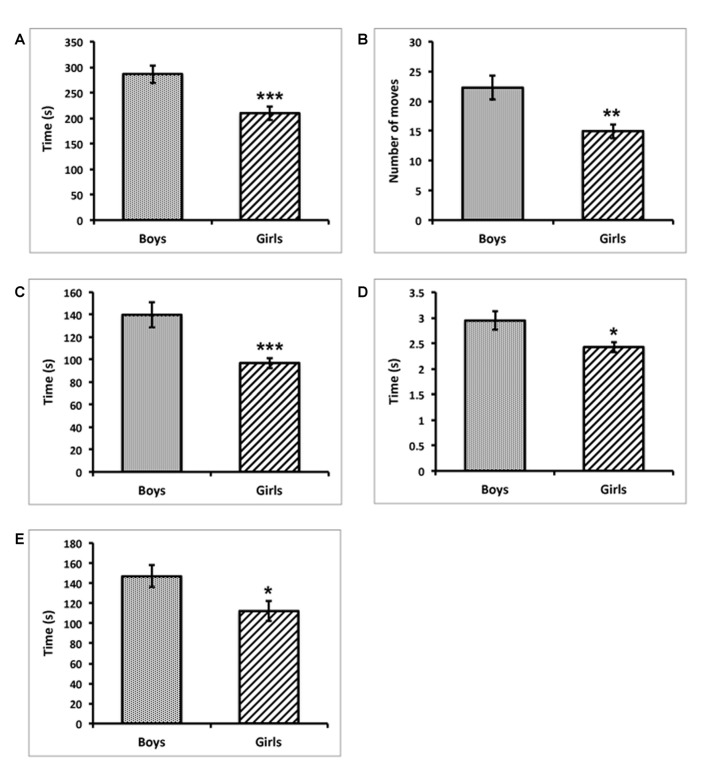
Girls were quicker than boys in time to solve puzzles (**A**; ***p < 0.001), made fewer wrong moves (**B**; **p < 0.01), spent less time moving pieces around overall (**C**; ***p < 0.001), spent less time on average moving individual pieces around the tablet (**D**; *p < 0.05) and watched the puzzle for a shorter time (**E**; *p < 0.05). All results represent the mean ± SEM.

Data analysis revealed that in the testing, girls outperformed boys in jigsaw puzzle solving, mainly due to the different strategy. As revealed by Bonferroni multiple comparison tests, girls were significantly quicker in solving each of the three puzzles (*F*_(1,45)_ = 12.51, *p* < 0.001; *η*^2^ = 0.22; Figure 2A), and made significantly less wrong moves (*F*_(1,45)_ = 9.92, *p* < 0.01; *η*^2^ = 0.19; Figure 2B) in the test. Girls also spend less time moving the puzzle pieces around the tablet (*F*_(1,45)_ = 13.67, *p* < 0.001; *η*^2^ = 0.24; Figure 2C), and average time of moving piece around the tablet was lower in girls than in boys (*F*_(1,45)_ = 6.69, *p* < 0.05; *η*^2^ = 0.13; Figure 2D). There was also a statistically significant difference in total time spend watching the puzzle (without moving or holding the pieces), but this difference was smaller, although still statistically significant (*F*_(1,45)_ = 5.40, *p* < 0.05; *η*^2^ = 0.11; Figure 2E). Qualitative observation of each subject confirmed the quantitative data, suggesting differences in the strategy. Namely, girls were in general more careful than boys when choosing a piece and placed it directly in the correct position while boys spend more time trying to fit the piece without careful observation where the piece might fit (trial and error attempts).

## Discussion

Sex differences in the brain and behavior are often the topic of heated debates. Although it is generally acknowledged that sex differences exist in certain behaviors, the origin of these differences remains unclear. There is still lot of discussions whether the sex differences in behavior and performance on different tasks are a consequence of biological/genetic differences or due to differences in childhood experiences (Miller and Halpern, 2014). Traditionally, even today, there are differences in upbringing of children of different sex. These range from subtle differences performed even by parents believing they do not perform differently towards sons or daughters, to differences in everyday consumer society, with toys and clothes, TV shows, cartoons and even adverts produced differently for girls and boys, what is today even more prominent than 20 or 30 years ago. Behaviors that show sex differences are usually very varied, not just between but also within sexes and are often clearly visible only when analyzing large cohorts (Miller and Halpern, 2014).

Although there are several references in the literature claiming that men are better than women in jigsaw puzzle solving, we could not find solid empirical evidence supporting these statements. Therefore, we analyzed sex differences in jigsaw puzzle solving in children in the present study. Results of our study revealed a robust sex difference with girls outperforming boys by a large margin. This difference was mainly due to different strategy towards solving the puzzle. Namely, the girls solved the puzzle more strategically. As observed qualitatively, and confirmed by quantitative data, the girls’ way to solve the puzzle was more focused. They choose the piece and place it in its proper location, while boys grabbed the piece and try to fit it without properly observing where the piece might fit looking at already solved parts of the puzzle. Girls thus solved the puzzle quicker, spent less time moving pieces around, and, most importantly, made fewer wrong moves (grabbing the piece and release it as it does not fit into the puzzle). The difference was robust, even with the small sample, there was highly statistically significant difference (*p* < 0.001), suggesting that the effect is real.

In the present study, all boys and girls were from the same school, from similar social and economical background, and it can be presumed that all originate from fairly emancipated families. Furthermore, all participants were average students and none of the had any diagnosed learning deficits or psychiatric disorders, although as the study was performed in young children, any psychiatric disorders or subtle learning deficits might not be evident yet and could therefore not be completely ruled out. However, perhaps the most important, at the time of the study none of the boys had been diagnosed or suspected of attention deficit disorder, which is more prevalent in boys than in girls and could potentially influenced the results, as such boys might have problems solving the jigsaw puzzle task. Therefore, an observed sex difference does suggest a difference in solving strategies between girls and boys that is unlikely to be influenced by differences in social or economic background, although in the future, similar task will have to be tested on different and larger populations to validate these data.

However, it has to be mentioned that our study was performed on a fairly small sample, the participants were not individually tested for any subtle psychological or learning deficits and their IQs were not assessed prior to the study, so there are possible confounders that might influence the results of the study. Therefore, to further confirm results from this study, larger studies will be needed in the future. On the other hand, in neither group there were significant outliers that would suggest large individual variability in groups of boys or girls that might skew the results.

Some of the most consistent sex differences are reported in visuospatial performances. In general, men usually score higher on mental rotation tasks and in tests measuring spatial perception and orientation (Voyer et al., 1995; Halpern et al., 2007). On the other hand, women usually score better on tests for memory location and spatial arrays (Ecuyer-Dab and Robert, 2004). Although there are many references in the literature about sex differences in solving jigsaw puzzles, we could not identify scientific articles reporting such differences, with the exception of the report by McGuiness and Morley (1991) who did not find sex differences in two-dimensional tasks, but found sex-differences in three-dimensional tasks. A recent study by Hoffman et al. (2011) has shown sex differences in solving a very simple, four piece puzzle in rural community in India, and this study suggested that sex differences in the ability to solve this puzzle is connected with the type of society, as sex difference was present only in patrilineal, but not in matrilineal society. Although this study is interesting, it also attracted some criticisms in design (Bailey et al., 2012), and it is also not directly relevant to our study as our study was performed in children, who were all familiar with solving jigsaw puzzles, and puzzle was more complicated (i.e., 25 vs. 4 pieces). Interestingly, in the literature, the references to sex differences in jigsaw puzzle solving suggest that boys outperform girls on those tasks (Caplan and Caplan, 1997; Helgeson, 2016). However, this is not supported by scientific literature and it is presumably derived from the general assumption that boys are better in visuospatial abilities than girls. In contrast, our study shows that girls were better than boys on this task, primarily due to deployment of a different strategy. The girls in our study appeared to be more systematic in solving the task, and have thus performed better than boys. The origin of this sex difference is yet unknown, but it would be very interesting to perform similar tasks in specific populations like patients with adrenal hypoplasia congenital (CAH), or twins of mixed sex, to see if there are any correlation between prenatal testosterone exposure and the strategy in solving jigsaw puzzle task.

In conclusion, our study reveals a robust sex difference in solving jigsaw puzzle. Contrary to certain notions (but not scientific reports) in the literature (Caplan and Caplan, 1997; Helgeson, 2016), girls outperformed boys on this task by a large margin, and it seems that this difference is much larger than men usually outperform women in different mental rotation tasks. For example, a very large multi-country study on sex difference in mental rotation tasks showed about 10% better performance of males vs. females (Lippa et al., 2010), while in our study the difference between girls and boys was much larger (50%). These results will have to be further validated, but they are strong indication of robust sex difference in the visuospatial ability to solve the jigsaw puzzle task.

## Author Contributions

VK designed the computer application, helped analyzing the results and writing the manuscript. MH performed part of the study and helped writing the manuscript. GM designed the study, performed part of the study and wrote the manuscript draft.

## Conflict of Interest Statement

The authors declare that the research was conducted in the absence of any commercial or financial relationships that could be construed as a potential conflict of interest.
